# Involvement of microRNA in Solid Cancer: Role and Regulatory Mechanisms

**DOI:** 10.3390/biomedicines9040343

**Published:** 2021-03-29

**Authors:** Ying-Chin Lin, Tso-Hsiao Chen, Yu-Min Huang, Po-Li Wei, Jung-Chun Lin

**Affiliations:** 1 Department of Family Medicine, School of Medicine, College of Medicine, Taipei Medical University, Taipei 110, Taiwan; 2 Department of Family Medicine, Wan Fang Hospital, Taipei Medical University, Taipei 116, Taiwan; greening1990@gmail.com; 3 Division of Nephrology, Wan Fang Hospital, Taipei Medical University, Taipei 116, Taiwan; 88128@w.tmu.edu.tw; 4 Department of Surgery, School of Medicine, College of Medicine, Taipei Medical University, Taipei 110, Taiwan; 5 Division of Gastrointestinal Surgery, Department of Surgery, Taipei Medical University Hospital, Taipei Medical University, Taipei 110, Taiwan; yuminhuang26@gmail.com; 6 Division of Colorectal Surgery, Department of Surgery, Taipei Medical University Hospital, Taipei Medical University, Taipei 110, Taiwan; 7 Cancer Research Center, Taipei Medical University Hospital, Taipei Medical University, Taipei 110, Taiwan; 8 Translational Laboratory, Department of Medical Research, Taipei Medical University Hospital, Taipei Medical University, Taipei 110, Taiwan; 9 Graduate Institute of Cancer Biology and Drug Discovery, Taipei Medical University, Taipei 110, Taiwan; 10 School of Medical Laboratory Science and Biotechnology, College of Medical Science and Technology, Taipei Medical University, Taipei 110, Taiwan; 11 Program in Medical Biotechnology, College of Medical Science and Technology, Taipei Medical University, Taipei 110, Taiwan; 12 Pulmonary Research Center, Wan Fang Hospital, Taipei Medical University, Taipei 110, Taiwan

**Keywords:** microRNA, solid cancer, post-transcriptional regulation

## Abstract

MicroRNAs (miRNAs) function as the post-transcriptional factor that finetunes the gene expression by targeting to the specific candidate. Mis-regulated expression of miRNAs consequently disturbs gene expression profile, which serves as the pivotal mechanism involved in initiation or progression of human malignancy. Cancer-relevant miRNA is potentially considered the therapeutic target or biomarker toward the precise treatment of cancer. Nevertheless, the regulatory mechanism underlying the altered expression of miRNA in cancer is largely uncovered. Detailed knowledge regarding the influence of miRNAs on solid cancer is critical for exploring its potential of clinical application. Herein, we elucidate the regulatory mechanism regarding how miRNA expression is manipulated and its impact on the pathogenesis of distinct solid cancer.

## 1. Introduction

Activation of tumorigenic processes mediate the neoplasia of normal cells, potentially resulting in initiation of malignancy. Exploration of regulatory mechanism relevant to the occurrence or progression of diverse cancers can be subjected to clinical application, such as early prevention, precise screening, or personal treatment. Among the regulatory factor involved in altered gene expression associated with carcinogenesis, the influence of microRNAs (miRNAs) has been widely pursued for decades [1]. MiRNA is a 22 nt-long noncoding RNA that functions as a post-transcriptional regulator for fine-tuning the coding efficiency of messenger RNA (mRNA) [2]. Targeting of the RNA-induced silencing complex (RISC) composed of single- strand miRNA, Argonaute (AGO), and GW182 (also known as TNRC6A) mediates the translational repression or degradation of the. regulatory candidate [3]. miRNA-mediate regulation is relevant to diverse cellular or environmental stress, including starvation, oxidative stress, hypoxia, and DNA breakdown, thereby being implicated in malignant disease [4]. Accordingly, dysregulation of miRNA expression is documented to exhibit bidirectional impact toward oncogenesis or tumor suppression [5].

**Figure 1:**
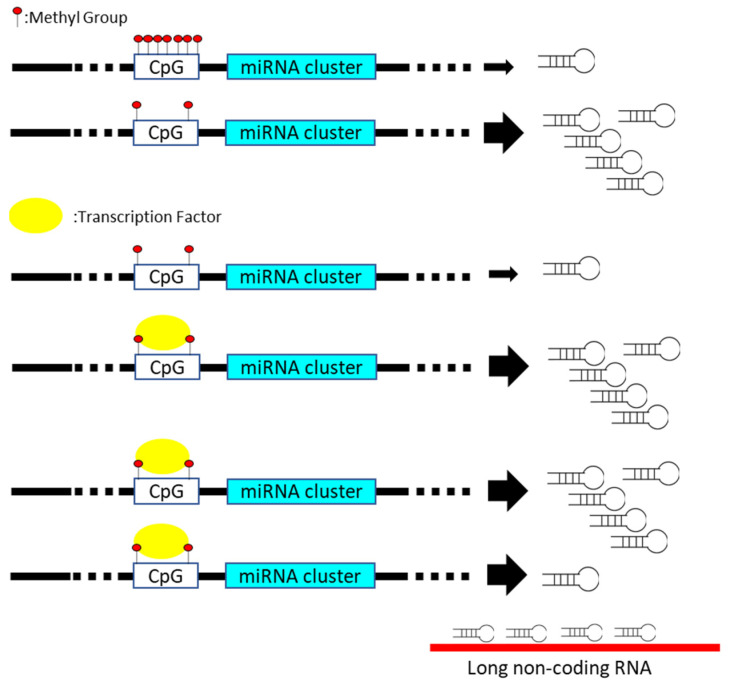
miRNA gene expression is manipulated via distinct mechanisms, including methylation (upper), transcription regulator (middle), and miRNA sponge (lower) in cancer cells.

Disturbance in miRNA expression can be induced via multiple steps, including transcriptional regulation, epigenetic methylation of miRNA-containing loci, miRNA processing pathway, and sequestration with long non-coding RNA, which functions as the miRNA sponge (Figure 1) [6,7]. For instance, various p53-responsive miRNAs networks containing miR-34 or miR-27b is documented to mediate the quiescence of distinct cancer cells [8,9]. The presence of mutant p53 reversely diminishes the tumor suppressive influence of p53-regulated miRNAs on carcinogenic signature [10]. Recent studies disclose the correlation between miRNA expression and epigenetic control regarding the methylation of CpG island within promoter region in cancer [11]. The silence of miR-127, miR-124-1, or miR-129-2 is closely related to the hypermethylation of CpG island-containing promoter in various solid cancer [12,13,14]. In addition, the function or expression of miRNA processing machinery, such as Drosha or the DGCR8 protein, is frequently deregulated in diverse malignancies [15]. Even though the impact of Drosha or DGCR8 on carcinogenesis is controversial, the disturbance of miRNA processing machinery is closely related to the global change in the miRNA expression profile [16]. The mutations of *Dicer* gene lead to DICER1 syndrome, which is relevant to the incidence of multiple cancers in an individual [17]. The biogenesis of miRNAs and gene expression profiles is disrupted with the mutant Dicer protein [18]. Recent studies document that miRNA loci are frequently annotated within the chromosomal regions, which are susceptible to the cancer-associated copy number variation (CNV) [19]. Cancer-mediated genomic instability results in the amplification or deletion of miRNA loci, subsequently leading to variation in the miRNA copy number [20].

According to the evidence gathered to date, we summarize the current knowledge regarding the impact of miRNA on the pathogenesis of frequent solid cancer, including colorectal cancer, lung cancer, breast cancer, and liver cancer. This review additionally evaluates the advantage or challenge associated with miRNA-based application in cancer treatment.

## 2. MiRNA and Solid Cancer

### 2.1. Colorectal Cancer

Colorectal cancer (CRC) is identified as the second leading cause of cancer-associated deaths worldwide with its high mortality and an increase in incidence [21]. In spite of progress in early screening, diagnosis, or prognostic prediction toward the incidence of CRC, miRNA is considered to be a potential biomarker for the assessment of CRC progression [22]. Decreases in the expression of miRNA clusters are frequently identified with the occurrence or development of CRC [23]. In the following section, the impact of several miRNA clusters on CRC are addressed.

### 2.2. Tumor-Suppressive miRNA in CRC

#### 2.2.1. Clusters miR-1/133a and miR-206/133b

The miR-1-1/133a-2, miR-1-2/133a-1, and miR-206/133b clusters are transcribed from distinct host genes. The influence of these miRNAs on the development of striated muscle has been widely documented [24]. Hypermethylation of the promoter within the host gene results in the decrease in the expression of miR-1/133 cluster in CRC tissues [25]. Moreover, miR-133 and 206 are specifically sponged by the complementary long non-coding RNA (lncRNA) ABHD11-AS1, XIST, and multiple LINC RNAs [26,27,28]. With the inhibitory effect toward the carcinogenic signatures, miR-1, miR-133a, and miR-206 are considered to be the tumor suppressors targeting a wide range of specific candidates. The in vitro results indicate the repressive effect of miR-1 on proliferation, migration, motility, and metabolism of CRC cells by targeting vascular endothelial growth factor (VEGF), notch receptor 3 (NOTCH3), and hypoxia-inducible factor 1 subunit alpha (HIF1A) gene [29,30,31]. The presence of miR-133 is demonstrated to diminish the growth or motility of CRC cells by targeting multiple candidates, such as fascin actin-bundling protein 1 (FSCN1) and oncogenic SUMO-specific peptidase 1 (SENP1) gene [32]. Upregulated miR-206 is relevant to reduced migration, proliferation, and immortality of CRC cells via targeting formin-like 2 (FMNL2), NOTCH3, and BCL2 gene [33].

#### 2.2.2. Clusters miR-15a/16-1 and miR-15b/16-2

miR-15 and miR-16 are encoded from two paralogues, miR-15a/16-1 and miR-15b/16-2 in the human genome. In CRC cells, an increase in the sirtuin 1 (SIRT1) protein mediates the down-regulated activity of promoter, which drives the transcription of miR-15b/16-2 cluster [34]. In addition, the mature miR-15/16 is sequestered by upregulated sponge LINC RNAs in distinct CRC cells [35,36]. Both in vivo and in vitro studies demonstrate the tumor-suppressive effect that is attributed to the presence of miR-15/16 clusters in CRC [37,38]. miR-15 and miR-16 are documented to target common candidates, including cyclin B1 and transcription factor AP-4, which majorly participated in the epithelial mesenchymal transition (EMT) [39]. Exogenous expression of miR-15 is reported to suppress the growth of CRC cells by targeting pro-survival BCL2 protein [40]. The presence of overexpressing miR-16 mediates the decrease in mortality and growth of CRC cells via suppressing the expression of KRAS proto-oncogene, GTPase (KRAS) protein both in vivo and in vitro [41]. Moreover, the reverse correlation between miR-16 level and VEGF receptor or MYB proto-oncogene is relevant to the prognosis of CRC patient [42].

#### 2.2.3. Clusters miR-100/let-7a/miR-125 and miR-99/let-7c

miR-100/let-7a/miR-125 and miR-99/let-7c are separately transcribed from an independent host gene in human genome but categorized in the same family with the related function. Nevertheless, the regulatory mechanism contributes to the alteration of the abovementioned miRNA clusters in CRC cells or tissues not comprehensively disclosed. For example, the abundance of sponge lncRNA is reversely relevant to the level of let-7a in CRC model [43]. The expressions of miR-125a/125b are manipulated through a complex process, including transcriptional control, hypermethylation-regulated epigenetic regulation, or sequestration with lncRNAs [44,45]. Nevertheless, the decreases in these miRNAs are frequently identified in CRC patients with poor prognosis [46]. An increase in the let-7 members mediates the cell cycle arrest and diminished cell growth via targeting of PHD and ring finger domains 2, Rho effector rhotekin, insulin-like growth factor 1, or MYC genes [47,48,49]. Upregulation of let-7c or let-7e lessens the metastatic activity of CRC cells through targeting the candidates encoding matrix metallopeptidase 11, PBX homeobox 3, and double cortin-like kinase 1 protein [50,51]. Furthermore, the impact of the let-7 family member on the abovementioned candidates sensitizes the CRC cells toward the chemo- or radiotherapy [52]. The expression profiles of miR-99a/99b are relevant to the level of the mechanistic target of rapamycin kinase (MTOR) protein in CRC cell lines [53]. Overexpression of the miR-125 family member facilitates the apoptosis of CRC cells by targeting the related factor, such as BCL2, BCL2 family members like 12, and myeloid cell leukemia 1 (Mcl-1) gene [54]. Moreover, an increase in miR-125a level leads to the suppression of angiogenic or metastatic activity of CRC cells by targeting VEGFA, SMAD-specific E3 ubiquitin protein ligase 1, and cAMP-responsive element-binding protein 5 gene [55,56].

### 2.3. Lung Cancer

Lung cancer (LC) is the leading cause of cancer-associated deaths worldwide, resulting in over 1 million deaths annually [57]. It is classified into two subsets according to the pathological signature—small cell lung cancer and non-small cell lung cancer. The complex mechanism involved in tumor initiation or progression is not comprehensively elucidated, which impedes the application of gene-based screening. A growing body of evidence indicates the association between the occasion of LC and the altered miRNA expression, which can be divided into oncomiR and tumor-suppressive miRNAs.

#### 2.3.1. Oncogenic miRNAs in LC Cells

MiRNA is widely considered a pivotal regulator in the control of cells growth [58]. With an increase in MYC expression, mis-regulated amplification of human miR-17-92 cluster, composed of six miRNAs, is noted in various solid tumor, including LC. An increase in the miR-17 cluster frequently leads to upregulated cell proliferation by targeting antiapoptotic factors, including transcription factor E2F1 or Phosphate and tensin homolog (PTEN) protein [59]. Immortality is a hallmark of cancerous cells, which is closely correlated with the decrease in p53 protein as previously illustrated [8,9]. In LC cells, the transcription of miR-34 family members is directly interfered with by the reduced p53 expression [60]. The cell cycle is subsequently disturbed by the augmentation of cyclin E2 and cyclin-dependent kinase, which is targeted by the miR-34 family in normal cells [60]. In contrast, the ectopic expression of miR-125b or miR-504 is both demonstrated to target p53 gene and in turn lessens the apoptotic sensitivity of LC cells toward environmental stress [61,62]. Metastasis of cancerous cells constitutes a predominant cause, which leads to the majority of cancer deaths [63]. Lung cancer is frequently diagnosed with the formation of metastases in the brain, bones, liver, and adrenal glands [64]. Activation of metastasis is associated with expression profiles of internal factors as well as external regulators involved in EMT process [65,66]. An increase in miR-10b level functions as a major factor attributed to active metastasis or enhance other oncogenic signatures of NSCLC cells [67,68].

#### 2.3.2. Tumor-Suppressive miRNAs in LC

Angiogenesis functions as a critical process toward the initiation and growth of solid tumor [69]. Throughout the process, the expression of VEGF and related regulation in response to hypoxia, such as the Akt/eNOS pathway, constitutes a crucial mechanism [70]. The presence of overexpressing miR-128 is demonstrated to exert repressive influence toward the phosphorylation of PI3K and p38 MAPK signaling, in turn lessening the levels of VEGFA, VEGF receptor (VEGFR) 2, and VEGFR3 [71]. The suppressive effect of miR-206 on angiogenic activity of NSCLC is achieved by targeting STAT3, HIF-1, or VEGF pathway [72]. The suppressive impact of miR-135a on IGF-1 expression further mediates the decreases in angiogenesis-related factors, including VEGF, bFGF, and IL-8 protein in A549 cells [73]. The decreases in various miRNAs mediate the loss of the tumor-suppressive effect on metastasis of LC. For instance, the restoration of miR-126 generation consequently inhibits the metastatic activity of NSCLC cells via targeting the chemokine receptor 1 gene [74]. Targeting of the ectopically expressing miR-192-5p on the TRIM44 gene is highly relevant to the reduced metastasis of LC cells, which is associated with the inactivation of the AKT/mTOR signaling pathway [75]. A decrease in miR-7-5p lessens its effect on repressing expression of NOVA2, which acts as an important regulator participating in angiogenesis and growth of NSCLC [76]. miR-206 exerts its tumor-suppressive impact on NSCLC metastases via targeting the actin-binding protein coronin 1C gene, which mediates the growth and metastasis in other solid cancers as well [77]. Overexpression of the miR-335 leads to reduction of the TGFβ-mediated EMT process in NSCLC by downregulating the level of ROCK1 gene, which plays an activator role in the PI3K/AKT/FAK pathway [78]. The presence of the miR-98 interferes with the translational activity of the TGFβR1 gene, subsequently diminishing the proliferation, migration, and invasion in distinct LC cell lines [79]. In contrast, the let-7 family or miR-126 is demonstrated to exert a suppressive impact on the proliferation of LC cells [80]. Exogenous expression of let-7 family member is reported to diminish proliferation of the LC cells via targeting the ras gene [81]. In NSCLC cells, the presence of miR-126 is relevant to the downregulated activity of PTEN/PI3K/AKT signaling, which critically control the cell growth [82].

### 2.4. Breast Cancer

Breast cancer (BC) is classified as the major cause in terms of high morbidity and mortality in women worldwide [83]. Obesity is often related to an aberrantly high level of estrogen, estrogen receptor (ER), progesterone receptor (PR), or human epidermal growth factor receptor (HER2), which constitutes the predominant mechanism for the initiation and progression of BC. Accordingly, miRNA involved in the regulation of adipogenesis is considered to be a potential factor to BC development [84]. In addition to CRC, mis-regulated expression miRNA cluster is frequently identified in BC [85].

#### 2.4.1. Oncogenic miRNAs in BC

An increase in this miR-183/96/182 cluster, which is located at 7q32.2, is identified in a variety of malignancies, including BC [86]. Bi-directional function of the miRNA cluster for oncogenesis or tumor suppressor is documented with the results of functional assays. Nevertheless, the alteration of this cluster is related to various cell process, such as apoptotic response, DNA repair, metabolism, or EMT process [87]. The transcription of the miR-183/96/182 cluster is activated with the increases in ZEB1 (zinc finger E-box binding homeobox 1) and HSF2 (heat shock transcription factor 2) protein in BC [86]. Moreover, activation of the HIF1 or PI3K/Akt pathway constitutes an additional mechanism attributed to the upregulation of the miR-183/96/182 cluster [88]. A growing body of study demonstrates that the presence of miR-183 manipulates the expression profiles composed of 45 genes in BC, including Programmed Cell Death 4, Early Growth Response1, Integrin Subunit Beta 1, p21, and p27, which is relevant to the abovementioned cell processes [89,90]. The presence of upregulated miR-96 facilitates the proliferation and migration of BC cells via targeting the protein tyrosine phosphatase, non-receptor type 9 gene [91]. Moreover, increases in the miR-96 and miR-182 synchronously interfere with the translation of FOXO1 protein, which maintains the homeostasis of glucose metabolism in normal lineages [92]. The members of the miR-183/96/182 cluster are incidentally noted in the exosome collected from the BC patients. Taken together, the miR-183/96/182 cluster mainly functions as the oncogenic factor toward the development of BC.

The miR-221/222 cluster is transcribed from Xp11.3 to encode two homologous miRNA members. The increases in miR-221/222 are highly relevant to the active cell proliferation, EMT process, and metastatic activity in BC cells via targeting a variety of tumor suppressors, including Transcriptional Repressor GATA Binding 1, Adiponectin Receptor 1, Suppressor of Cytokine Signaling 1, Cyclin Dependent Kinase Inhibitor 1B, ERα, p27, and TIMP Metallopeptidase Inhibitor 3 genes [93]. Upregulation of miR-221/222 leads to the transformation of ER-α positive tumors to ER-α negative BC by targeting the ER-α gene [94]. Moreover, the aberrant expression of the miR-221/222 cluster is advantageous to self-renewal of the BC stem cell self by targeting the PTEN-Akt signaling pathway [95]. Taken together, the miR-221/222 acts as a predominant oncogene in the origin of ER-negative BC with the aggressive signature.

#### 2.4.2. Tumor-Suppressive miRNAs in BC

The miR-199a/214 cluster is annotated at chromosome1q24 to generate miR-199a-5p, miR-199a-3p, and miR-214 transcripts. The hedgehog-signaling Vitamin D Receptor and miR-214 are demonstrated to constitute a cross-talk axis involved in the downregulation of miR-199/214 expressions in BC cells [96]. The decrease in the miR-199/214 cluster results in the loss of its tumor-suppressive effect in triple negative BC (TNBC). For instance, depletion of the miR-199a/214 cluster induces the EMT-like phenotype in normal cell lineage [85]. Overexpression of the miR-199a/214 cluster reversely lessens the proliferative activity of TNBC cells by targeting hedgehog signaling, which is proposed as a potential therapeutic strategy toward BC [96]. The H3K27me3-related epigenetic silencing is associated with the levels of the Enhancer of zeste homolog 2 (Ezh2) protein, which induces neoplasia in various tissues. The inverse correlation between the miR-199/214 and Ezh2 or Ki-67 protein is identified by using an in vitro BC cell model [97]. The impact of miR-199/214 on interfering with the generation of Ezh2, β-catenin, or Ki-67 constitutes another mechanism for diminishing the proliferative activity of BC cells [98,99]. Accordingly, the tumor-suppressive influence of miR-199/214 cluster on inhibiting proliferation, migration, and invasion of BC cells is documented with majority of the present studies.

#### 2.4.3. Oncogenic and Tumor-Suppressive of miR-23/27/24 Cluster

Two paralogs of this cluster, miR-23a/27a/24-2 and miR-23b/27b/24-1, are respectively annotated in chromosomes 19 and 9. The results of functional studies suggest that the transcribed member of the cluster executes both oncogenic and tumor-suppressive effects on the carcinogenic signature of BC cells. Upregulated transcription of miRNA-23/27/24 induces the progression of BC cells via targeting the Hypermethylated in Cancer 1 gene, which functions as a repressor to tumor growth [100]. Nevertheless, the impact of miR-23b and miR-27b targeting on tumor growth is controversial, based on the in vitro cultured or in vivo nude mice model [101]. Moreover, the members of this cluster share a variety of candidates, such as Sprouty RTK Signaling Antagonist 2, BCL2 Antagonist/Killer, PPARγ, and Nischarin gene [102]. This miRNA cluster also exerts its influence by collaborating with other miRNAs on BC development. For instance, FOXO1 expression is coordinatively regulated by the miRNA-23/27/24 cluster along with miR-27a, miR-96, or miR-182 in BC [92]. In contrast, targeting of miR-27a to the Transmembrane Protein 170B gene is demonstrated to suppress the Wnt/β-catenin pathway, in turn lessening the proliferative and migratory efficiency of BC cells [103]. Moreover, targeting of the overexpressing miR-23b-3p to PAK2 and phosphorylation of the myosin light chain II gene subsequently reduces the metastatic activity of BC cells in vitro [104]. Taken together, the comprehensive insight into the manipulation of miRNA-23/27/24 expression and its coordination with other miRNA is useful in controlling BC development.

### 2.5. Liver Cancer

Hepatocellular carcinoma (HCC) is identified as the third cause of cancer mortality in the Asia-Pacific region, which consequently accounts for over 80% of liver cancers [105]. In addition to viral infection, aflatoxin-contaminated food-, alcohol-, or obesity-mediated cirrhosis, the impact of miRNA is demonstrated in the context of HCC [106]. With its potential to manipulate gene expression profiles, miRNA is practicable as a novel therapeutic or emerging biomarker for the stratification of HCC patient.

#### 2.5.1. Oncogenic miRNAs in HCC

miR-21 is a well-characterized oncomiR in a variety of solid tumors, including HCC [107]. An increase in miR-21 level with the incident of HCC is consistently revealed by using in vitro and in vivo experiments [108]. Aberrant expression of miRNA-21 with a concomitant decrease in tumor-suppressive PTEN protein leads to activation of focal adhesion kinase, Akt, and mTOR signaling, in turn resulting in active cell metastasis and proliferation [109]. Activation of a series kinase cascade is associated with the increase in MMP-2 and MMP-9, which further strengthens the pathogenesis and development of HCC [109]. An elevated level of miR-21 in plasma potentially provides high accuracy toward the screening or diagnosis of early-stage HCC [110].

miR-221 is identified as another oncomiR with an upregulated level in HCC [111]. By directly targeting the cell cycle inhibitors, including CDKN1B/p27 and CDKN1C/p57, the influence of miR-221 on active progression of HCC is consistently disclosed in multiple studies [112]. Direct targeting of miR-221 to the DNA damage-inducible transcript 4 gene relieves its tumor-suppressive activity in HCC model [111]. Moreover, the presence of miR-221 strengthens the anti-apoptosis activity of HCC cells by manipulating the level of the Bcl2 modifying factor protein (BMF), the pro-apoptotic member of the Bcl-2 family [113]. In contrast, inhibition of miR-221 increases the susceptibility of HCC to apoptotic stimuli with an elevated level of BMF and the downstream caspase pathway [113].

#### 2.5.2. Tumor-Suppressive miRNAs in HCC

miR-29 family is comprised of four members, miRNA-29a, miRNA-29b-1, miRNA-29b-2, and miRNA-29c, which are transcribed from chromosomes 7q32.3 and 1q32.2. An miRNA profiling study first identified the downregulation of miR-29 in LC patients associated with poor prognosis and survival rate [114]. By using an in vitro cultured system, activation of TGF-β or nuclear factor kappa B (NF-κB) drives the downregulation of miRNA-29, in turn facilitating the expressions of extracellular matrix genes in hepatic stellate cells [115]. miR-29 family members are disclosed to share a variety of candidates, including CDC42, PIK3R1, Bcl-2, and Mcl-1 gene [116]. In line with these results, targeting of endogenous Bcl-2 and Mcl-1 gene by miR-29 family member sensitizes the HepG2 cells to apoptosis-induced chemotherapeutic treatment or serum starvation [116]. In addition to miR-29, the inverse correlation between Mcl-1 and miR-101 or miR-125b is also identified in liver tumor cells [117,118]. Similarly, respective targeting of miR-101 or miR-125b share the same effect on sensitizing the liver cancer cells with the presence of apoptotic treatment via targeting Mcl-1 gene. miR-122 is the most abundant miRNA, which fine-tunes a variety of cellular processes in hepatocyte [119]. The transcription of miRNA-122 is controlled by the expression of liver-specific factors, including CCAAT/enhancer-binding protein (C/EBP) and hepatocyte nuclear factor (HNF) family members [120]. A decrease in miRNA-122 level associated with hepatocarcinogenesis, active metastasis, and poor prognosis is identified in HCC tissue [121]. The presence of miRNA-122 exerts the tumor suppressive impact on HCC development via targeting cyclin G1, pyruvate kinase isoform M2, and Wnt family member 1 gene [112]. Th miR-122/cyclin G1 axis facilitates the stability of p53 and therefore promotes the sensitivity of HCC cells to doxorubicin-induced apoptosis [112].

### 2.6. Ovarian and Cervical Cancer

Ovarian cancer and cervical cancer is ranked as the first and fourth cause of cancer death in women worldwide [122,123], respectively. Around 65% of ovarian cancer is classified as surface epithelial according to the World Health Organization (WHO) classification [124]. Even though understanding the biological signature of ovarian cancer was gradually revealed with the recent progress and knowledge, the severity or mortality of ovarian cancer remains unchanged for the last 30 years. A promising biomarker, such as the cancer-specific miRNA, toward early screening or precise diagnosis of ovarian cancer is crucial. On the other hand, the majority of cervical cancer is initiated by the infection of certain subtypes of the human papilloma virus (HPV) [125]. The relevance of HPV infection with the chronic inflammation that subsequently mediates the initiation of cervical carcinogenesis remains controversial. Recent studies document that the aberrant miRNAs profile is identified throughout the initiation and development of cervical cancer [126]. Moreover, the altered miRNA level exhibits influence on manipulating the carcinogenic process of cervical cancer.

#### 2.6.1. Oncogenic miRNA in Ovarian Cancer and Cervical Cancer

Metastasis of ovarian cancer is modulated via the interplay between miRNA and signaling factors involved in the EMT pathway [127]. An increase in miR-17-5p level is relevant to the active progression and EMT activity of ovarian cancer cells by targeting PTEN expression and downstream signaling [128]. In contrast, the administration of an miR-17-5p inhibitor interferes with the migration and invasion activity of ovarian cancer cells by using in vitro cultured assays [128]. The presence of miR-214 targets the PTEN expression, which in turn reduces the sensitivity of in vitro cultured ovarian cancer cells to cisplatin [129].

In cervical cancer, a growing body of studies demonstrate the oncogenic influence of miR-21 with the identification of diverse targets, including *PDCD4*, *PTEN*, *TIMP-3*, *TNF-α,* and *ANXA1* genes [130,131,132]. Therefore, upregulated miR-21 is highly related to the active inflammation and metastasis of cervical cancer cells. The upregulated level of miR-155 in peripheral blood and tissues collected from cervical cancer patients has been revealed in recent studies. The presence of miR-155 targets the expression of SOSC1, which in turn enhances progression and inflammation in cervical cancer [133].

#### 2.6.2. Tumor-Suppressive miRNA in Ovarian Cancer and Cervical Cancer

A decrease in miR-150 is frequently identified in epithelial ovarian cancer cells [134]. The presence of overexpressing miR-150 lessens the invasive and metastatic activity of ovarian cancer cells by targeting the expression of Zinc Finger E-Box Binding Homeobox 1 (ZEB1) protein [135]. miR-150 is suggested as a potential therapeutic target for intervening the metastasis of ovarian cancer [135]. Similarly, the upregulation of miR-22, miR-183, or miR-31 level is reported to result in reduced migration or invasion of serous ovarian carcinoma by interfering with the expression of the TIAM1 protein [136]. Moreover, overexpressing miR-7 directly targets the expression of EGFR protein, which leads to reversion of the EMT signature in ovarian cancer through AKT and ERK1/2 pathways [137].

Multiple miRNAs exert a suppressive effect on the chronic inflammation, which is crucial for the development of cervical cancer [138]. Downregulation of miR-429 is relevant to the active inflammation in cervical cancer tissues with IL-6 and IFN-β production, which is driven through the NF-κB pathway [139]. IKKβ (the primary kinase toward NF-κB activation) is identified as a new target of miR-429 in cervical cancer cells [139]. miR-101 is documented to exert a tumor-suppressive impact on the proliferation, invasion, and inflammation of cervical cancer cells by directly targeting COX-2 protein [140]. The reverse association of the high mobility group box 1 (HMGB1) with miR-34a, miR-1284, and miR-142 is identified in the cervical cancer cells [141,142,143]. HMGB1 is a well-characterized oncogene that is involved in chronic inflammation, progressive tumorigenesis, active metastasis, and therapy resistance of cervical cancer tissues. Downregulation of miR-24, miR-451, let-7a, and miR-125a is noted as well in cervical cancer, which is relevant to the active inflammation. An increase in chitinase-3-like protein 1 with a concomitant decrease in miR-24 is proposed to facilitate the proliferation, metastasis, and inflammation in cervical cancer [144]. The tumor-suppressive impact of miR-451 on lessening the inflammation, invasion, angiogenesis, and proliferation of cervical cancer cells is demonstrated by directly targeting the expression of the IL-6 receptor [144]. The generation of HPV oncoprotein E6 mediates the decreases in let-7a and miR-125, in turn relieving the suppressive effect of let-7a and miR-125 on STAT3 expression [145,146]. The presence of STAT3 further facilitates expression of the HPV E6 protein through transcriptional regulation [145]. Taken together, STAT3 and HPV E6 constitute a feed-forward circuit that participates in the downregulation of let-7a and miR-125 throughout the development of cervical cancer. Reversely, the complementation of miR-125 leads to the decreases in STAT3, MMP-9, MMP-2, and N-cadherin levels and activities, subsequently diminishing the proliferation, metastasis, and inflammation of cervical cancer cells [146].

## 3. Role of Exosomal miRNA and Its Application

Exosomes are extracellular vesicles ranging from 30 to 150 nm in size that can be secreted by normal or cancer cells [147]. Secretion of exosome is demonstrated to deliver a messenger, including protein or miRNA, between normal and cancer cells [148]. It is documented that cancer cells secrete 10-fold more exosomes than that of normal cells, which is critical to the recruitment and development of carcinogenic environment [149]. Taking lung cancer as an example, miR-96-containing exosomes secreted from H1299 cells were demonstrated to exhibit oncogenic activity toward upregulated cell proliferation by directly targeting the production of the LIM-domain only protein 7 (LMO7) expression [150]. The presence of exosomal miR-23a secreted by lung cancer cells was documented to facilitate tumor angiogenesis under both normoxia and hypoxia conditions, suggesting that the genetic messenger was transmitted from lung cancer cells to distant endothelial cells [151]. Furthermore, the drug resistance of lung cancer cells is closely related to the existence of exosomal miRNA. A recent study reported that the gemcitabine-resistant A549 (A549-GR) cells assembled miR-222-3p-containing exosomes, which were transmitted into parental gemcitabine-sensitive cells and subsequently promoted their migration, invasion, and gemcitabine resistance by targeting the expression of the SOCS3 protein [152]. Additionally, the transfer of the miR-21-containing exosome assemble by gefitinib-resistant H827R cells to HCC827 cells activated AKT signaling and lead to gefitinib resistance of parental gefitinib-sensitive cells [153]. 

Taken together, exosomal miRNA is considered an ideal tool for diagnosis as well as therapeutic targets with its influence on the carcinogenic pathway or environment. As for lung cancer cells, five exosomal miRNAs, including miR-205, miR-19a/19b, miR-30b, and miR-20a, were considered the diagnostic markers of squamous cell lung carcinoma (SQCLC) with their decreases in the circulating levels after surgery [154]. Three SQCLC-related miRNAs, including miR-10b-5p, miR-15b-5p, and miR-320b, were demonstrated to be promising biomarkers with area under the ROC curve (AUC) values between 0.936 and 0.911 toward diagnosis of the disease [155]. In addition to diagnosis or early prediction, exosome is demonstrated as an ideal vehicle for drug delivery within recent studies [156]. For instance, the usage of engineered exosomes containing miR-21 sponge was reported to mediate a decrease in miR-21 in U87-MG glioma cell lines, in turn lessening its carcinogenic signature by relieving the miR-21-mediated suppression on the expression of PDCD4 and RECK protein [156]. Nevertheless, the problems of miRNA-mediated influence, such as off-target phenomenon, is not ignored with the promising reports.

## 4. Conclusions and Perspectives

MiRNAs are involved in diverse cell process to maintain the homeostasis of normal cells through complex network (Table 1 and Table 2). This phenomenon makes aberrant miRNA profiles interesting biomarkers for initiation or progression of solid tumor as well as potential targets for precise treatment. Nevertheless, precise targeting or site-specific delivery of solid cancer-specific miRNA could be a major impediment in the use of miRNA-based therapy. In addition, a better knowledge regarding the off-target effect and comprehensive assessment of toxicity is another critical concern to be solved. In this review, we summarize the present knowledge of miRNA-mediated influence on the carcinogenic signature of frequent solid tumor, which may highlight a potential opportunity for clinical translation and potential application.

## Figures and Tables

**Table 1:** The impact of classified oncomiR on the carcinogenic signature through the specific target.

miRNA	Disease	Manipulating Mechanism	Candidate	Physiological Influence	Reference
miR-17	LC; Ovarian cancer	CNVs; Transcriptional control (MYC)	E2F1, PTEN	Cell Growth, apoptosis, metastasis	[55,128]
miR-125b	LC	Epigenetic control	p53	Apoptosis	[60]
miR-504	LC	Transcriptional control (EGFR signaling)	p53	Apoptosis	[61]
miR-10b	LC	Epigenetic control	Homeobox D10,	Metastasis	[71,72]
miR-183	BC	Transcriptional control (ZEB1, HSF2)	PCD4, EGR1, p21, p27	Apoptosis, DNA repair, metabolism, EMT	[84,85,86]
miR-96	BC	miRNA sponge	FOXO1, PTPN9	Proliferation, migration, metabolism	[87]
miR-182	BC	miRNA sponge	FOXO1	Metabolism	[88]
miR-221/222	BC	Epigenetic control, miRNA sponge, transcriptional control (TGF-β)	Transcriptional Repressor GATA Binding 1, Adiponectin Receptor 1, Suppressor of Cytokine Signaling 1, Cyclin Dependent Kinase Inhibitor 1B, ERα, p27, and TIMP Metallopeptidase Inhibitor 3	Proliferation, EMT process, metastasis	[89]
miR-23/27/24	BC	Transcriptional control (HIC1)	Hypermethylated in Cancer 1 (HIC1), Sprouty RTK Signaling Antagonist 2, BCL2 Antagonist/Killer, PPARγ, Nischarin, Transmembrane Protein 170B, PAK2	Cell growth, migration	[88,99,100]
miR-21	HCC; Cervical Cancer	miRNA sponge	PTEN, MMP2, MMP9, PDCD4, PTEN, TIMP-3, TNF-α, ANXA1	Metastasis and proliferation	[105,130,131,132]
miR-221	HCC	Transcriptional control (NF-κB)	CDKN1B/p27, CDKN1C/p57 DNA damage-inducible transcript 4, BMF	Cell growth, apoptosis	[108,109]
miR-214	Ovarian Cancer	Transcriptional control (hedgehog signaling)	PTEN	Metastasis, chemoresistance	[129]
miR-155	Cervical Cancer	miRNA sponge, transcriptional control (c-MYB)	SOSC1	Inflammation	[133]

**Table 2:** The impact of classified tumor-suppressive miRs on carcinogenic the signature through the specific target.

miRNA	Disease	Manipulating Mechanism	Candidate	Physiological Influence	Reference
miR-1	CRC	Epigenetic control; miRNA sponge	VEGF, NOTCH3	Proliferation, migration, motility and metabolism	[25,26,27]
miR-133	CRC	Epigenetic control; miRNA sponge	FSCN1, SENP1	Growth or motility of CRC cells	[28]
miR-206	CRC, LC	Epigenetic control; miRNA sponge	FMNL2, NOTCH3, BCL2, STA3, HIF-1, Coronin 1C	Migration, proliferation, and immortality, metastasis	[29,65,76]
miR15/16	CRC	Transcriptional control (SIRT1)	cyclin B1, TFAP-4, Bcl-2,K-Ras, MYB	Epithelial mesenchymal transition (EMT), apoptosis	[35,36,37,38]
let-7 family	CRC; Cervical cancer	miRNA sponge	PHD, ring finger domains 2, RTKN, IGF-1, MYC, MMP11, PBX3, DCLK1, STAT3	Cell cycle arrest, metastasis	[43,44,45,46,47,145,146]
miR-125	CRC Cervical Cancer	miRNA sponge	Bcl-2, Mcl-1, SMURF1, VEGFA, CREB5, STAT3, MMP-9, MMP-2, N-cadherin	Apoptosis, angiogenic or metastatic activity, inflammaation	[50,51,52,146]
let-7 family	LC	miRNA sponge	Ras	Proliferation	[57]
miR-126	LC	miRNA sponge	PTEN, CX3CR1	Proliferation, metastasis	[58,73]
miR-34	LC; Cervical Cancer	Transcriptional control (p53)	Cyclin E2, HMGB1	Cell cycle arrest	[59,141]
miR-128	LC	miRNA sponge	VEGFA, VEGFR2, VEGFR3	Angiogenesis	[64]
miR-135a	LC	miRNA sponge	IGF-1	Angiogenesis	[66]
miR-192	LC	Transcriptional control (p53)	TRIM44	Metastasis	[74]
miR-7	LC; Ovarian cancer	miRNA sponge	Nova2, EGFR	Angiogenesis, EMT	[75,137]
miR-335	LC	miRNA sponge	ROCK1	EMT	[77]
miR-98	LC	miRNA sponge	TGFβR1	Proliferation, migration, and invasion	[78]
miR-199	BC	Transcriptional control (hedgehog signaling)	Ezh2, β-catenin, Ki-67	Proliferation, migration, and invasion	[93,94,95]
miR-214	BC	Transcriptional control (hedgehog signaling)	Ezh2, β-catenin, Ki-67	Proliferation, migration, and invasion	[93,94,95]
miR-29	HCC	Transcriptional control (NF-κB, TGF-β)	CDC42, PIK3R1, Bcl-2, Mcl-1	Cell cycle, apoptosis	[112]
miR-101	HCC; Cervical Cancer	miRNA sponge	Mcl-1, Cox-2	Apoptosis, inflammation, proliferation, invasion	[115,140]
miR-125b	HCC	Epigenetic, transcriptional control; miRNA sponge	Mcl-1	Apoptosis	[115]
miR-122	HCC	Transcriptional control (C/EBP, HNF)	Cyclin G1, PKM2, and Wnt family member 1	Cell cycle, apoptosis	[108]
miR-150	Ovarian cancer	miRNA sponge	ZEB1	Invasion, metastasis	[134,135]
miR-22, miR-183, miR-31	Ovarian cancer	Transcriptional control (Snail)	TIAM1	Invasion, migration	[136]
miR-429	Cervical Cancer	miRNA sponge	IL-6,IFN-β	Chronic inflammation	[139]
miR-142	Cervical Cancer	miRNA sponge	HMGB1	Chronic inflammation, progressive tumorigenesis, active metastasis	[141,142,143]
miR-24	Cervical Cancer	Uncertain	chitinase-3-like protein 1	Proliferation, metastasis, inflammation	[144]
miR-451	Cervical Cancer	Uncertain	IL-6 receptor	Inflammation, invasion, angiogenesis, proliferation	[144]

## Data Availability

Not applicable.
